# Monitoring extracellular vesicle surface glyco-properties using fluorescent lectins and nanoparticle tracking analysis

**DOI:** 10.1098/rsob.250136

**Published:** 2025-11-26

**Authors:** Ninoslav Mitic, Filip Janjic, Jelena Danilovic-Lukovic, Sanja Goc, Tamara Jankovic, Miroslava Jankovic

**Affiliations:** ^1^ Department for Immunochemistry and Glycobiology, University of Belgrade – Institute for the Application of Nuclear Energy – INEP, Belgrade, Serbia; ^2^ Department of Life Sciences, University of Belgrade – Institute for Multidisciplinary Research, Belgrade, Serbia

**Keywords:** extracellular vesicles, fluorescence nanoparticle tracking analysis, ultracentrifugation, size exclusion chromatography, filtration, lectins

## Abstract

Extracellular vesicles are small particles released by all cell types. Different extracellular vesicle isolation methods are widely used, yet none achieve an optimal balance between yield, purity and structural integrity. This study aimed to establish a comparative approach for evaluating different extracellular vesicle preparations using nanoparticle tracking analysis. A simple one-step assay relying on fluorescence-based nanoparticle tracking analysis was used to evaluate lectin binding to extracellular vesicles as a measure of possible changes in their surface glycosylation during various isolation methods. Seminal extracellular vesicles were isolated from normozoospermic men using ultracentrifugation alone—UC-sEVs—or combined with size exclusion chromatography—UC-SEC-sEVs—or microfiltration—UC-MF-sEVs. They were analysed based on their size and lectin-binding properties using wheat germ agglutinin and *Ricinus communis* agglutinin I. While total seminal extracellular vesicles and tetraspanin-positive seminal extracellular vesicles maintained similar size distributions across all isolates, lectin-positive seminal extracellular vesicles displayed a shift towards larger than 200 nm seminal extracellular vesicles in UC-SEC-sEVs and UC-MF-sEVs, as compared to UC-sEVs. The ratio of larger (>200 nm) to smaller (30–200 nm) lectin-positive sEVs was increased, particularly for wheat germ agglutinin in UC-MF-sEVs and *Ricinus communis* agglutinin I in UC-SEC-sEVs. These findings demonstrate that size exclusion chromatography and microfiltration combined with ultracentrifugation influence seminal extracellular vesicle surface glycosylation and alter lectin binding across extracellular vesicles of different sizes.

**Figure:**
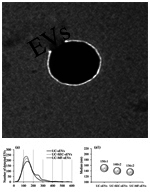

## 1. Introduction

Extracellular vesicles (EVs) are small, spherical particles with a distinctive bilayer membrane, released by all cell types into the extracellular space and body fluids. They are suggested to play key roles in intercellular communication due to their ability to transport a diverse range of bioactive molecules, including (glyco)proteins and nucleic acids [1,2]. EVs have been increasingly studied over the past decade and are recognized as a valuable resource for biomarker discovery and potential drug delivery systems [3].

Different methods for EV isolation based on their biophysical and biochemical properties are currently in use or under development [4,5]. However, no optimal balance between EV yield and purity in terms of separation of specific contaminants as well as structural integrity, all of which could influence analysis of EV function or EV-associated biomarkers, has been met in any of them [4,6]. Taken together, all these issues interfere with EV standardization as a prerequisite for their biomedical applications.

This study was aimed at establishing an approach for comparison of different EV preparations, based on nanoparticle tracking analysis (NTA), extending its application beyond estimation of size and concentration of EVs. For the first time, a simple one-step assay relying on fluorescence-based NTA (F-NTA) was used to evaluate lectin binding to EVs as a measure of the possible changes in their surface glycosylation during various isolation steps. Thus, F-NTA allows real-time visualization of particles in suspension depending on the binding of different labelled probes/molecules to the surface of EVs only. In this way, it is advantageous over other assays, such as solid-phase-based ones, since the possible contribution of soluble and any other contaminants that can be found in EV preparations to the binding is overcome.

Monitoring surface properties of EVs including glycan compositions and presentations, during isolation and purification (from the same or different sources), is of special importance for their use in functional assays and for therapeutical applications [7–9]. Thus, the EV membrane is known to protect cargo molecules from degradation, present specific molecules for targeting recipient cells and carry molecules that modulate immune responses, i.e. participate in signalling interactions [10,11].

Examination was performed on EVs from seminal fluid (sEVs), where prostasomes (prostate-derived EVs) are the most abundant. They were isolated from samples of normozoospermic men using ultracentrifugation (UC) alone or in combination with size exclusion chromatography (SEC) or microfiltration (MF), as the most available methods which are commonly used in EV research. Lectin binding to sEVs was used to compare preparations obtained by these methods, relying on EV size. However, SEC and MF may differently impact the EV surface through shear forces and other mechanical stresses, potentially causing membrane deformation or the removal of loosely bound proteins from EV surfaces [12–14]. Based on previous results on surface glycosylation of prostasomes [15–17], wheat germ agglutinin (WGA) (sialic acid/*N*-acetylglucosamine (GlcNAc)-binding lectin) was used as the lectin of choice. In addition, *Ricinus communis* agglutinin I (RCA I) (specific for Gal or GalNAc), which has non-overlapping specificity to WGA, was used to address polylactosamine structures, known to be enriched on the EV surface. Selected surface glycans were monitored across two sEV size ranges: 30−200 and >200 nm. Size is one of the parameters currently used for EV classification. Specifically, EVs are classified based on their size into small (<200 nm) EVs and large (>200 nm) EVs [18]. We refer to this classification because all three methods used for sEV isolation are size-based, and it aligns with previous reports defining ‘true’ prostasomes (sEVs) as those within the 30−200 nm range [18,19].

The results obtained, together with previous data on glycans as markers of prostasome populations and distinct membrane domains [15,20], highlight, in general, surface glycans as a rich source of information for EV profiling. Their relevance should be considered when designing experiments, depending on the intended application in EV research.

## 2. Material and methods

### 2.1. Materials

Rabbit recombinant monoclonal anti-TSG101 antibody (clone EPR7130 (B)) was from Abcam (Cambridge, UK) and mouse monoclonal anti-CD63 antibody (clone TS63) and mouse monoclonal beta-actin antibody (clone AC-15) were from Invitrogen (Waltham, MA, USA). Biotinylated goat anti-rabbit IgG, biotinylated goat anti-mouse IgG, fluorescein isothiocyanate-conjugated plant lectins WGA and RCA I and an Elite Vectastain ABC kit were from Vector Laboratories (Burlingame, CA, USA). Fluorescently labelled antibodies anti-human CD63 Alexa Fluor^®^ 488 (clone MEM-259) and anti-human CD9 R-phycoerythrin (PE) (clone MEM-61) were from EXBIO (Vestec, Czech Republic). GlcNAc, galactose (Gal) and bovine serum albumin (BSA) were from Sigma (St Louis, MO, USA). A silver stain kit and Clarity Western ECL Substrate were from Bio-Rad (Hercules, CA, USA). Unstained Protein Standards, Broad Range (10–200 kDa), were from New England Biolabs GmbH (Frankfurt, Germany). Superdex™ 200 and Amersham Protran 0.45 nitrocellulose membrane were from Cytiva (Marlborough, MA, USA). Polyethersulfone (PES) 0.2 µm syringe filter Filtropur S was from Sarstedt (Nümbrecht, Germany). All other chemicals were p.a.

#### 2.1.1. Human semen samples

In this study, pre-existing and anonymized human semen samples, collected for routine analysis, were used, and as such, the study is not classified as research involving human subjects. Aapproval (no. 02-1462/2) was obtained from the institutional ethics committee in accordance with guidelines aligned with the 1975 Helsinki Declaration (revised 2013). Sperm parameters such as number, morphology and motility were evaluated in accordance with the established criteria outlined by the World Health Organization (2021, 6th edition). Sperm cells and debris were removed from the ejaculate using centrifugation at 1000*g* for 20 min.

### 2.2. Methods

#### 2.2.1. Isolation of extracellular vesicles from human seminal plasma

Three different procedures (A–C) were used for isolation of EVs from a single pool of human seminal plasma (SP) from normozoospermic men. The pool consisted of 15 individual SP samples (1 ml each).

(A) UC: SP pool was differentially centrifuged at 10 000*g* (30 min) and 100 000*g* (60 min) on an Optima L-90K ultracentrifuge (Beckman Coulter, Indianapolis, IN, USA). The final pellet, enriched in sEVs, was washed at 100 000*g* (60 min), resuspended in 1 ml of 0.05 M Tris–HCl buffer, pH 7.6, and divided into three equal parts (P-1, P-2 and P-3). P-1 was additionally washed at 100 000*g* (60 min), resuspended in 100 µl of 0.05 M Tris–HCl buffer, pH 7.6, designated as UC-sEVs and used for further analysis.
(B) UC and SEC: P-2 was subjected to gel filtration on Superdex™ 200, following the protocol of Carlsson and colleagues, which was modified to include CD63-immunoreactivity as an EV indicator [17,21]. Immunoreactive fractions were pooled, centrifuged at 100 000*g* (60 min), and the pellet was resuspended in 100 µl of 0.05 M Tris–HCl buffer, pH 7.6. It was designated as UC-SEC-sEVs.
(C) UC and MF: P-3 was resuspended in 50 ml of 0.05 M Tris–HCl buffer, pH 7.6, to allow filtration through a 0.2 µm filter. The filtrate was centrifuged at 100 000*g* (60 min), and the pellet was resuspended in 100 µl of 0.05 M Tris–HCl buffer, pH 7.6. It was designated as UC-MF-sEVs.

#### 2.2.2. Transmission electron microscopy

Transmission electron microscopy was performed as previously described [10]. On formvar-coated copper grids, 200 mesh, 10 µl of sEV sample was applied by grid flotation. Binding took place at room temperature for 45 min. After that, fixation with 2% formaldehyde was performed for 10 min, followed by washing three times with particle-depleted 0.05 M phosphate buffer saline (PBS), pH 7.2 (dPBS), for 2 min. Post-fixation was performed with 2% glutaraldehyde for 5 min, followed by rinsing with deionized water for 5 min. The grids were dried at room temperature, after which images were captured using a Philips CM12 electron microscope (Philips, Eindhoven, The Netherlands).

#### 2.2.3. Sodium dodecylsulfate–polyacrylamide gel electrophoresis

Samples were separated on 10% acrylamide gel [22]. Proteins were stained using a silver staining kit, following the manufacturer’s instructions (Bio-Rad). The gel was calibrated using unstained broad-range sodium dodecyl sulfate–polyacrylamide gel electrophoresis protein standards.

#### 2.2.4. Immunoblot

After electrophoresis was performed under reducing conditions for TSG101/beta-actin detection and non-reducing conditions for CD63 detection, proteins were transferred onto a nitrocellulose membrane by a Tank Transfer System (Bio-Rad) using transfer buffer, 0.025 M Tris containing 0.192 M glycine and 20% methanol, pH 8.3, under a constant voltage of 100 V for 60 min. The membrane was blocked with 1% BSA in 0.05 M PBS, pH 7.2, for 120 min at room temperature. Rabbit monoclonal anti-TSG101 antibody (0.458 μg ml^−1^), mouse monoclonal anti-CD63 antibody (0.25 μg ml^−1^) or mouse monoclonal anti-beta-actin antibody (1 : 1000) diluted in 0.5% BSA/PBS, pH 7.2, was used for immunoblot and incubated overnight at 4°C. Following the washing steps, corresponding biotinylated secondary antibodies, avidin/biotinylated horseradish peroxidase mixture from the Elite Vectastain ABC kit, and Clarity Western ECL Substrate solution were used following the manufacturer’s guidelines. The immunoreactive bands were detected using a ChemiDoc MP Imaging System (Bio-Rad Laboratories, Hercules, CA, USA).

#### 2.2.5. Nanoparticle tracking analysis

The size distribution and median size of corresponding sEV isolates were analysed using a ZetaView Quatt PMX-430 nanoparticle tracking analyser, with ZetaView software v. 8.05.16 SP3 (Particle Metrix, Inning am Ammersee, Germany). Following an automatic cell check, the camera and laser were aligned, and appropriate focus was verified using 100 nm polystyrene beads, as per the manufacturer’s instructions. sEVs were diluted in dPBS to achieve optimal particle counts per frame (150–180). sEV isolates were exposed to a blue laser (488 nm) during measurements conducted in light scatter mode. A washing step was done between each measurement using dPBS. For video acquisition, a shutter speed of 100 and a frame rate of 30 throughout one cycle were used, with a sensitivity set to 78. Post video-capturing parameters were configured to a minimal area of 10, maximal area of 1000, and minimum brightness of 30.

Measurements of each sEV preparation (UC-sEVs, UC-SEC-sEVs and UC-MF-sEVs), derived from a single SP pool, were performed in triplicate (technical replicates) at up to 11 positions. Results were analysed using descriptive statistical analysis. Mean values and standard deviations were calculated using Microsoft Excel (Office 2019).

#### 2.2.6. Fluorescence-based nanoparticle tracking analysis

For F-NTA analysis, Alexa Fluor^®^ 488-conjugated anti-CD63 antibody (incubation dilution 1 : 320), R-phycoerythrin-conjugated anti-CD9 antibody (incubation dilution 1 : 200) and fluorescein-conjugated lectins WGA and RCA I (incubation concentrations of 0.5 and 1.25 µg ml^-1^, respectively) were used. sEVs and labelled antibodies/lectins were incubated in a 40 µl volume with dPBS for 2 h at room temperature. After that, the reaction mixture was filled to 1 ml with dPBS in order to obtain an optimal concentration of sEVs for F-NTA measurements (300 and 360 particle counts per frame, as recommended by the manufacturer). For the inhibition assay, fluorescein-conjugated lectins WGA and RCA I were preincubated with 100 mM GlcNAc or 100 mM galactose (Gal), respectively, followed by the same protocol as described above. For video acquisition, 488/520 nm laser and 500/550 nm cut-off filter were used, shutter speed was set to 200 and sensitivity was 95. Measurements of each labelled sEV preparation were performed in triplicate (technical replicates) at up to 11 positions. Results were analysed using descriptive statistical analysis. Mean values and standard deviations were calculated using Microsoft Excel (Office 2019).

The share (in percentage) of particles within each size range (30–200 nm and >200 nm) was calculated relative to the sum of all detected particles within the same sample. This was applied separately for total sEVs, as well as for tetraspanin- and lectin-positive sEVs. The contribution of small (30–200 nm) and large (>200 nm) particles to total lectin binding was expressed as the ratio of share of lectin-positive particles to share of total particles for each method of sEV isolation. This was further used for the introduction of the index value, which was calculated by dividing the contribution of large particles by the contribution of small particles. The relative index was obtained by dividing the UC-SEC-sEVs and UC-MF-sEVs indices by the UC-sEVs index (assigned a value of 1).

## 3. Results

**Figure 1.**
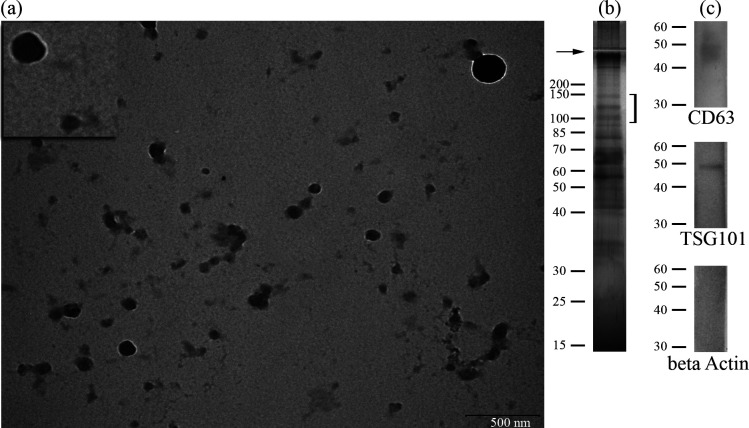
Common appearance of sEVs isolated by ultracentrifugation. (a) Representative transmission electron microscopy image showing size heterogeneity of sEV preparation. Part of the image is enlarged to better visualize cup-shaped morphology typically associated with EVs. Scale bar, 500 nm. (b) Electrophoretic pattern of sEVs comprising three characteristic protein bands (marked with bracket) known as the prostasome signature as revealed by silver staining. (c) Immunoblot confirmed the presence of common EV markers: CD63 (membrane) and TSG101 (cytosolic protein recovered in EVs: cargo), as well as the absence of beta-actin, an EV-negative marker. Numbers indicate the position of molecular mass standards (kDa). The presented data were obtained on one UC-sEVs preparation isolated from one pool of seminal plasma.

**Figure 2.**
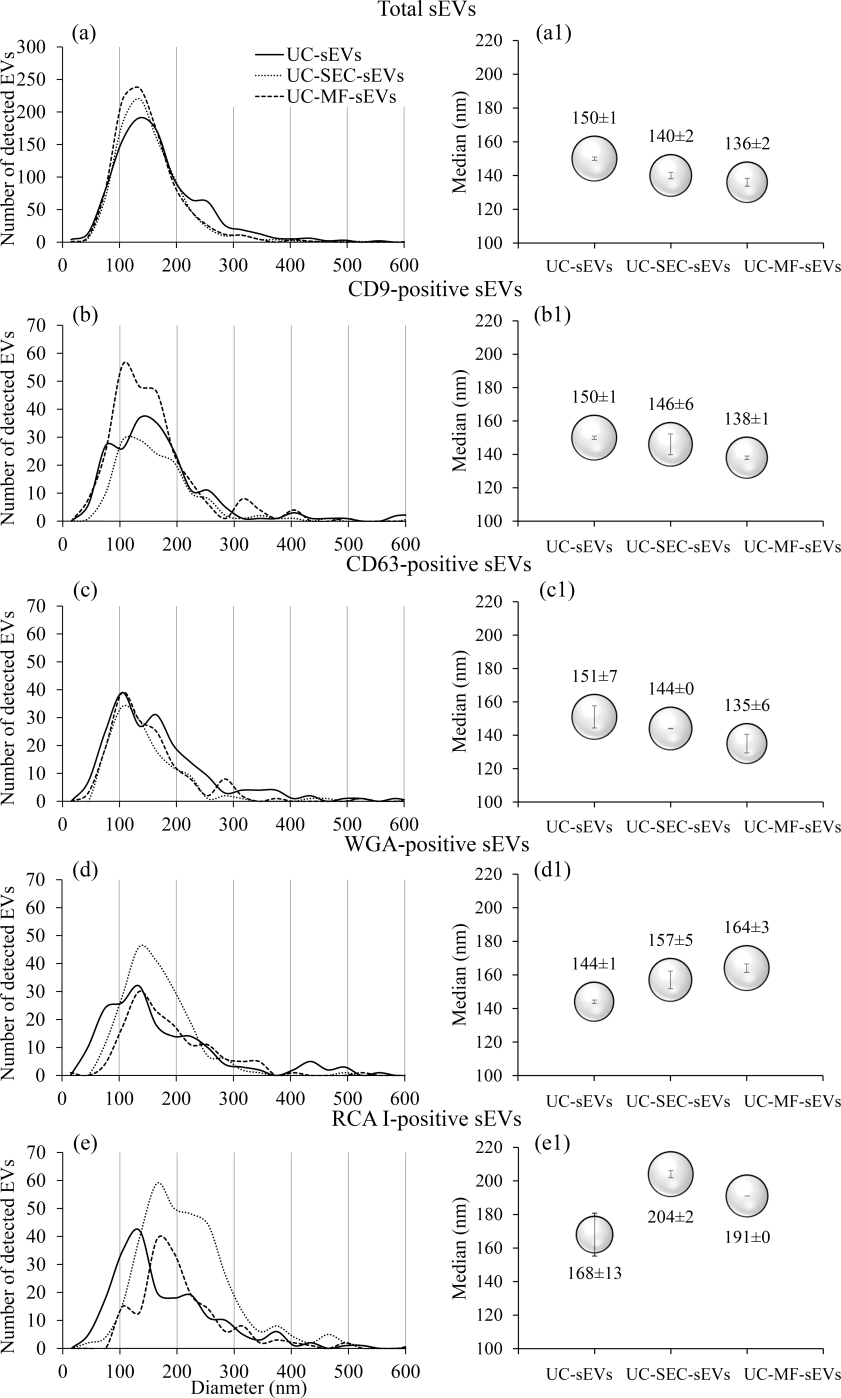
Size distribution and median diameters of sEV isolates. Representative size distribution of total (a), tetraspanin-positive (b,c) and lectin-positive sEVs (d,e) obtained by NTA. Graphical illustration of median diameters obtained by NTA: total (a1), tetraspanin-positive (b1,c1) and lectin-positive sEVs (d1,e1). The circle sizes are illustrative and based on their median diameters within each graph. NTA analysis of each sEV preparation (UC-sEVs, UC-SEC-sEVs and UC-MF-sEVs), derived from a single seminal plasma pool, was performed in triplicate (technical replicates). The size of the bars within the circles graphically represents the standard deviation. Median values displayed above the circles represent the mean ± standard deviation. NTA, nanoparticle tracking analysis; RCA I, *Ricinus communis* agglutinin I; sEVs, seminal EVs; UC-MF-sEVs, sEVs isolated by ultracentrifugation and microfiltration; UC-SEC-sEVs, sEVs isolated by ultracentrifugation and size exclusion chromatography; UC-sEVs, sEVs isolated by ultracentrifugation; WGA, wheat germ agglutinin.

sEVs (figure 1) isolated by UC (UC-sEVs) and those further subjected to SEC (UC-SEC-sEVs) or MF (UC-MF-sEVs) displayed comparable size distribution and median diameters (MD) for total sEVs and tetraspanin-positive sEVs (figure 2a–c,a1–c1). In contrast, the size distribution of lectin-positive sEVs in both UC-SEC-sEVs and UC-MF-sEVs shifted towards larger diameters compared to UC-sEVs (figure 2d,e), reflecting also in an increase in MD (figure 2d1,e1). Moreover, the MD of RCA I-positive sEVs was higher than that of WGA-positive sEVs, irrespective of the isolation method used (figure 2e1). The carbohydrate-mediated binding of both tested lectins was confirmed in a competitive sugar inhibition assay, demonstrating inhibition of their binding to all three sEV isolates (electronic supplementary material, figure S1).

To overcome the influence of intrinsic heterogeneity in size and particle number (due to the nature of EVs as an analyte) and to get insight into lectin binding to small and large sEVs, the analysis was based on a comparison of the share of distinct particles.

**Figure 3.**
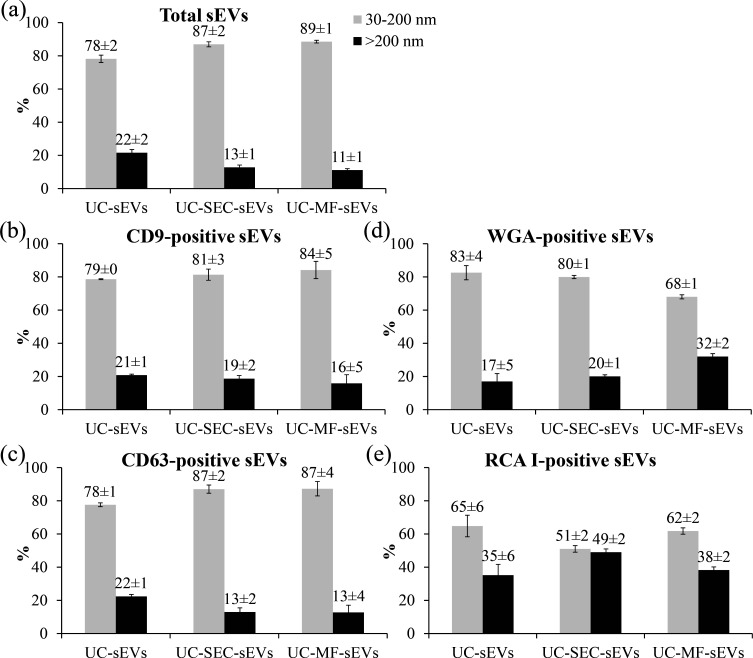
Monitoring of seminal EVs across different size distribution ranges. Shares (in percentage) of total (a), tetraspanin-positive: CD9 (b), CD63 (c) and lectin-positive: WGA (d), RCA I (e) are presented. The share of particles within each size range (30–200 nm and >200 nm) was calculated relative to the sum of all detected particles within the same sample, applied separately to total sEVs, lectin- and tetraspanin-positive sEVs. NTA analysis of each sEV preparation (UC-sEVs, UC-SEC-sEVs and UC-MF-sEVs), derived from a single seminal plasma pool, was performed in triplicate (technical replicates). Numbers above each column indicate mean ± standard deviation. Error bars visually depict the standard deviation. RCA I, *Ricinus communis* agglutinin I; sEVs, seminal EVs; UC-MF-sEVs, seminal EVs isolated by ultracentrifugation and microfiltration; UC-SEC-sEVs, seminal EVs isolated by ultracentrifugation and size exclusion chromatography; UC-sEVs, seminal EVs isolated by ultracentrifugation; WGA, wheat germ agglutinin.

Referring to two specified size ranges, the majority of sEVs was within the size range of 30−200 nm in all three sEV isolates. The shares of total sEVs in this size range were 78% for UC-sEVs, 87% for UC-SEC-sEVs and 89% for UC-MF-sEVs (figure 3a). Specifically, the shares of CD9-positive sEVs were 79% for UC-sEVs, 81% for UC-SEC-sEVs and 84% for UC-MF-sEVs (figure 3b). The shares of CD63-positive sEVs were 78% for UC-sEVs and 87% for both UC-SEC-sEVs and UC-MF-sEVs (figure 3c). By contrast, WGA- and RCA I-positive sEVs showed a lower share within this size range. WGA-positive sEV shares were 83% for UC-sEVs, 80% for UC-SEC-sEVs and 68% for UC-MF-sEVs (figure 3d). RCA I-positive sEV shares were 65% for UC-sEVs, 51% for UC-SEC-sEVs and 62% for UC-MF-sEVs (figure 3e). The share of sEVs which had a size higher than 200 nm was minor. Total sEV shares in this range were 22% for UC-sEVs, 13% for UC-SEC-sEVs and 11% for UC-MF-sEVs (figure 3a). The shares of CD9-positive sEVs were 21% for UC-sEVs, 19% for UC-SEC-sEVs and 16% for UC-MF-sEVs (figure 3b). CD63-positive sEV shares were 22% for UC-sEVs and 13% for both UC-SEC-sEVs and UC-MF-sEVs (figure 3c).

The share of large lectin-positive sEVs differed, especially for RCA I-positive sEVs. Large WGA-positive sEV shares were 17%, 20% and 32%, and RCA I-positive sEV shares were 35%, 49% and 38% for UC-sEVs, UC-SEC-sEVs and UC-MF-sEVs, respectively (figure 3d,e). Although all isolates showed increased RCA I-binding towards large sEVs, this was most pronounced in UC-SEC-sEVs, where RCA I-positive vesicles displayed a nearly equal distribution between the small and large EV size ranges. The observed differences in corresponding shares were consistent with the differences observed in the MD of tetraspanin- and lectin-positive sEVs.

Given the observed differences, the contribution of lectin-positive sEVs within the specified size range was further assessed. The contribution of WGA-positive sEVs in the 30−200 nm range was higher in UC-sEVs (1.1) compared to those >200 nm (0.8) (table 1). In contrast, for both UC-SEC-sEVs and UC-MF-sEVs, the contribution of WGA-positive sEVs larger than 200 nm was higher (1.6 and 2.9, respectively) compared to those in the 30−200 nm range (0.9 and 0.8, respectively).

**Table 1.** Lectin reactivity of small and large sEV isolates. Abbreviations: sEVs, seminal EVs; WGA, wheat germ agglutinin; RCA I, *Ricinus communis* agglutinin I; UC-sEVs, sEVs isolated by ultracentrifugation; UC-SEC-sEVs, sEVs isolated by ultracentrifugation and size exclusion chromatography; UC-MF-sEVs, sEVs isolated by ultracentrifugation and microfiltration.

	WGA			RCA I		
	UC-sEVs	UC-SEC-sEVs	UC-MF-sEVs	UC-sEVs	UC-SEC-sEVs	UC-MF-sEVs
contribution^a^ of small particles (A)	1.1	0.9	0.8	0.8	0.6	0.7
contribution of large particles (B)	0.8	1.6	2.9	1.6	3.8	3.4
index^b^ (B/A)	0.7	1.8	3.6	2.0	6.3	4.9
relative index^c^	—	2.6	5.1	—	3.2	2.5

Small particles: 30–200 nm; large particles: >200 nm.

^a^ A ratio of lectin-positive particle shares to total particle shares was calculated for each method and termed as contribution.

^b^ The index value was calculated by dividing the contribution of large particles by the contribution of small particles. The index quantifies how much large particles contribute compared to small particles, with values >1 indicating a higher contribution from large particles.

^c^ The relative index was introduced to allow comparison of lectin reactivity across isolates. It was calculated by dividing the index of either UC-SEC-sEVs or UC-MF-sEVs by the index of UC-sEVs (starting material). UC-sEVs served as the reference (assigned a value of 1).

A different trend was observed for RCA I-positive sEVs in UC-sEVs, where the contribution of large sEVs was higher than that of small ones (1.6 and 0.8, respectively). This was also seen in UC-SEC-sEVs (3.8 and 0.6) and UC-MF-sEVs (3.4 and 0.7). Overall, the contribution of larger relative to smaller sEVs varied depending on the isolation method (table 1). When UC-sEVs was used as a reference, the relative index was higher in UC-SEC-sEVs and UC-MF-sEVs, being most pronounced for WGA in UC-MF-sEVs (5.1) and for RCA I in UC-SEC-sEVs (3.2) (table 1).

## 4. Discussion

This study demonstrates that different EV isolation methods influence surface glycosylation of sEVs. Combining either SEC or microfiltration with ultracentrifugation alters lectin-binding preferences, shifting it towards sEVs larger than 200 nm.

Initially, we demonstrated that the size distribution and the MD of tetraspanin-positive sEVs corresponded to those of total particles across all isolates, whereas lectin-positive sEVs exhibited a different trend. Size distribution of sEVs was assessed in terms of shifts towards smaller or larger vesicles, as reflected in MD changes. Aside from WGA-positive sEVs in UC-sEVs, the MD of both WGA- and RCA I-positive sEVs was higher relative to total sEVs and was higher in additionally purified isolates compared to UC-sEVs. Moreover, MD of both lectin-positive sEVs is higher in additionally purified isolates compared to UC-sEVs. Similar findings were reported by Lennon and colleagues, where WGA staining of pancreatic cancer cell-derived EVs resulted in a higher apparent EV diameter [23]. The observed increase in lectin-positive sEV diameter suggests selectivity in lectin binding towards sEV populations with greater diameter; however, from these results, it remained unclear whether this shift was driven by sEVs below or above the 200 nm threshold.

Beyond differences in MD, an analysis of the sEV populations within two defined size ranges further emphasized the impact of isolation methods. The share of tetraspanin-positive sEVs matched that of total particles and, together with size distribution and MD results, verified the vesicular nature of particles in analysed sEV isolates. In contrast, despite the increased share of small sEVs in additionally purified isolates, the share of lectin-positive sEVs deviated from both total and tetraspanin-positive sEVs, indicating greater lectin binding preference towards large sEV population. This was most pronounced for WGA-positive sEVs in the UC-MF-sEVs isolate, whereas increased RCA I-binding to large sEVs, though observed across all isolates, was most notable in UC-SEC-sEVs.

These results also demonstrated that combining EV isolation methods influenced the share of small and large sEVs, increasing the share of the 30−200 nm sEV population, with no notable differences between UC-SEC-sEVs and UC-MF-sEVs. While MF was expected to remove most sEVs larger than 200 nm by retaining them in the filter retentate, SEC was anticipated to preserve the original ratio of large and small sEVs. The unexpected lower recovery of larger sEVs in SEC might be due to mild retention effects or subtle interactions with the column matrix.

To further compare additionally purified isolates with UC-sEVs and quantify differences in lectin binding, the contribution of large to small WGA- and RCA I-positive sEVs (index) within each isolate was analysed. This index was higher in both UC-SEC-sEVs and UC-MF-sEVs compared to UC-sEVs, following the same pattern observed earlier—an over fivefold increase for WGA in UC-MF-sEVs and an over threefold increase for RCA I in UC-SEC-sEVs. These findings indicated that both SEC and MF modulate lectin-binding preferences, affecting large and small sEVs differently, with MF predominantly affecting WGA binding, and SEC having a more pronounced influence on RCA I binding. One possible explanation for these observations lies in the impact of isolation methods on the EV surfaces. These surfaces consist of integral, peripheral and lipid-anchored membrane proteins, along with (glyco)proteins that adsorb onto EVs, at least in part, after their release [24,25], forming a protein corona. The corona comprises both tightly bound ‘hard corona’ proteins and loosely associated ‘soft corona’ proteins. Given that human SP is rich in highly glycosylated proteins [26–29], these ligands, as part of the EV soft corona, may remain present but with modified availability [23,30], potentially affecting lectin binding. Thus, variations in isolation methods, by selectively enriching sEVs and separating them from soluble glycoproteins, may alter the density and accessibility of specific ligands on sEV surfaces. Several studies have demonstrated that exosomes often carry distinct glycan motifs due to selective sorting during multivesicular body maturation, differing from the parent cell surface or plasma membrane-derived vesicles [8,31,32]. Lectin-based profiling further supports that surface glycans can serve as indicators of EV subpopulations based on their biogenesis route [33,34]. Thus, glycosylation analysis can improve EV subpopulation resolution and complement size and protein marker-based characterization.

Differences in the composition of soft corona across different seminal EV isolates could also significantly impact downstream functional assays. Recent studies indicated both the importance of EV protein corona, as well as that it can be disturbed by choice of EV isolation method [30,35,36]. It was reported that EVs with functional corona enhance angiogenesis, skin regeneration and immunomodulation and that removal of corona from EV surfaces by SEC or UC abrogates angiogenesis [36]. Findings of Singh and colleagues showed that differential UC and SEC strip the surface-associated proteins from EVs [37]. Our results showed preserved but altered sEV reactivity with WGA and RCA I after the application of SEC or MF, providing insight into the impact of these methods on the EV surface. Conversely, Nordin and colleagues demonstrated that, unlike UC, the combination of ultrafiltration with SEC does not alter vesicle outer protein composition and maintains the EVs biophysical and functional properties [38].

Our findings align with previous studies highlighting the role of glycosylation in EV–protein interactions. For example, glycan modifications have been linked to the selective recruitment of specific glycoforms to EV surfaces, as seen in studies of antithrombin binding [24]. The observed variations in lectin reactivity across sEV isolates further support the notion that glycan composition differs between sEVs of different sizes and is altered by isolation methods. Such differences may have functional implications for EV biodistribution, cellular uptake and interactions with other biogenic nanoparticles [24,33], as well as broader implications for EV glycosylation studies.

Although various methods have been used to characterize specific EV populations [13,23,39], direct comparisons of glycan composition among EV isolates remain scarce. Our findings demonstrate that EV isolation methods impact surface glycosylation, altering lectin binding across EVs of different sizes. This change in surface EV glyco-components may influence downstream analyses, as specific EV subsets could gain or lose functional properties relevant to their biological role. Given the role of glycans in EV interactions with recipient cells and their microenvironment, future studies relying on EV surface glyco-components should consider the impact of isolation methods on their composition, presentation and availability.

## Data Availability

All relevant data are available in the paper or in the supplementary material [40]. Any additional supporting data can be provided upon request.
